# High-Temperature Mechanical Behaviors of SiO_2_-Based Ceramic Core for Directional Solidification of Turbine Blades

**DOI:** 10.3390/ma13204579

**Published:** 2020-10-14

**Authors:** Jiangwei Zhong, Qingyan Xu

**Affiliations:** Key Laboratory for Advanced Materials Processing Technology (MOE), School of Materials Science and Engineering, Tsinghua University, Beijing 100084, China; zhongjw16@mails.tsinghua.edu.cn

**Keywords:** superalloy, ceramic core, high temperature, mechanical behavior, auxiliary thermal system

## Abstract

The high-temperature mechanical behaviors of SiO_2_-based ceramic cores for the directional solidification of turbine hollow blades were investigated. Isothermal uniaxial compression tests of ceramic core samples were conducted on a Gleeble-1500D mechanical simulator with an innovative auxiliary thermal system. The stress–strain results and macro- and micro- structures of SiO_2_-based ceramic cores were investigated experimentally. The microstructures were characterized by the scanning electron microscope (SEM). Based on the experimental data, a nonlinear constitutive model for high temperature compressive damage was established. The statistical results of Weibull moduli show that the stability of hot deformation increases with the increase of temperature. The fracture type of the SiO_2_-based core samples is brittle fracture, but when the temperature exceeds 1400 °C, the mechanical behavior exhibits thermo-viscoelastic and viscoplastic property. Under high-temperature (>1400 °C) and stress conditions, the strength of the ceramic core is weakened owing to the viscous slip of SiO_2_, which is initially melted at the temperature of 1400 °C. The comparison results between the predictions of nonlinear model and experimental values indicate that the model is applicable.

## 1. Introduction

In response to the increasing worldwide need for reliable, low-cost, and environmentally compatible generation of energy, the new generation of H-class gas turbines (GT) is developed [1,2]. Ni-based single-crystal (SX) superalloy turbine blades, which are the key hot-end assemblies of the gas turbine engines, can be produced by using the directional solidification (DS) technology [3]. The complex inner cavity formed by the ceramic core provides the possibility for the development of the hollow blade cooling technology. Nowadays, due to the complex thermal stress–strain interactions during DS, the size of blades appears imprecise, and the ceramic core even appears cracked. As a result, the performance of the SiO_2_-based ceramic core directly affects the dimensional accuracy of the SX hollow turbine blade. Therefore, the high-temperature mechanical properties of the SiO_2_-based ceramic core are crucial for the preparation of SX hollow turbine blade.

There are some studies focused on the high-temperature mechanical behaviors of SiO_2_-based ceramic cores [4]. Xu et al. [5] investigated the flexural strength of silica-based ceramic cores doped with different silica nanopowders at 1540 °C. The results showed that large quantities of cristobalite were crystallized at 1540 °C, which might provide enhanced mechanical property in the casting. Niu et al. [6] found that the ceramic cores with 3 wt % mullite fibers showed excellent properties, such as flexural strength being 22.3 MPa at 1550 °C, owing to fiber reinforcing. At the same time, there are two opposite conclusions about the high-temperature properties of SiO_2_-based ceramic core. For instance, Kazemi et al. [7] found that the increase of zircon content could result in the decrease of cristobalite formed in situ, owing to cristobalite crystallized on the surface of fused silica particles during heat treatment. This result is contrary to Wang and Hon’s report [8], but is in good agreement with the result of Wilson et al. [9]. On the other hand, there are many examples in the literature exploring the static and dynamic hot deformation behaviors of other types of ceramic materials in-depth, such as ZrB_2_ [10], SiC [11], Al_2_O_3_ [12], and ceramic composite material [13]. The methods presented in these articles can be applied to the study of SiO_2_-based ceramic core.

During the directional solidification process, the ceramic cores will be subjected to mechanical loading at high temperature for a long time. In order to prevent the core fracture, it is very necessary to investigate the high temperature behavior of ceramic core. In this study, an auxiliary thermal system is employed to carry out the thermal compression tests of ceramic core. Constitutive modeling and various characterization methods are used to understand the high-temperature mechanical property of SiO_2_-based ceramic core.

## 2. Experimental Procedure

### 2.1. Experimental Methods and Design

The characteristics of fused silica and zircon used as raw materials are illustrated in Table 1. According to the formulation used in actual production, the composition of the samples was 60 wt % fused silica and 40 wt % zircon. Porous silica-based ceramic cores were prepared by using ceramic injection molding. After a series of procedures, such as mixing, ball milling, adding adhesive, and drying, the green bodies were obtained. The sintered samples were subsequently subjected to heat treatment at 1500 °C for 30 min and then were removed from the furnace at 1000 °C, to the atmosphere at 25 °C, for simulating the realistic rapid cooling process during the directional solidification from the heating zone into the cooling zone.

**Figure 1:**
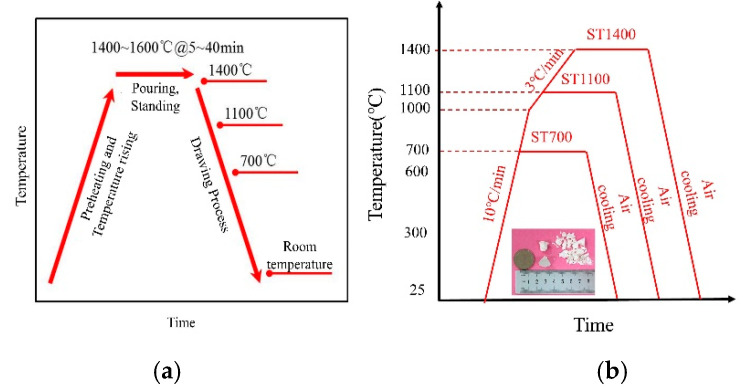
(**a**) Thermal process of directional solidification; and (**b**) the schematic diagram illustrating the compression process of SiO_2_-based ceramic cores.

Thermal process of ceramic shell/core during directional solidification is shown in Figure 1a. Since the tendency of core breakage is mainly concentrated at temperatures above 800 °C, the hot compression temperatures of ceramic cores were set at 700 °C (ST700), 1100 °C (ST1100), and 1400 °C (ST1400). The sample ST25 was tested at 25 °C. The average size of the ceramic cores is 14.77 mm in diameter and 15.25 mm in length (φ14.77 mm × 15.25 mm). The stain rate of 0.001 s^−1^ was chosen. After taking into consideration the inhomogeneity of the ceramic core, we tested three parallel samples for each deformation temperature. The whole process can be represented by Figure 1 and Table 2.

### 2.2. High-Temperature Experimental System for Mechanical Behaviors

**Figure 2:**
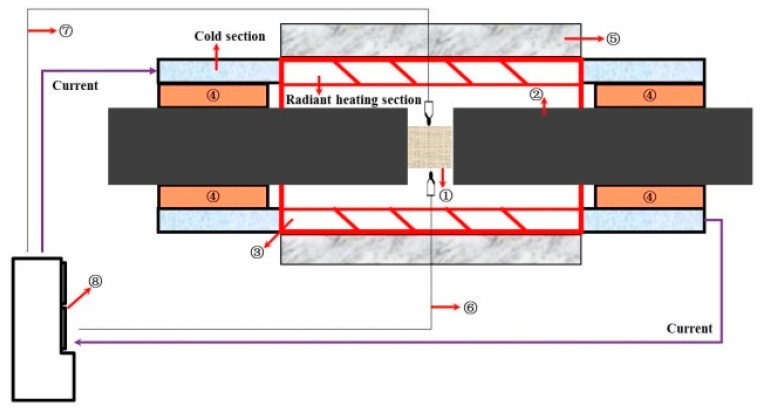
Schematic diagram of the auxiliary thermal system (① ceramic sample, ② compression bars, ③ silicon carbide screw tube, ④ corundum tube, ⑤ insulation fiber box, ⑥ S-type thermocouple for temperature control, ⑦ temperature S-type thermocouple for temperature calibration, and ⑧ temperature control cabinet).

The Gleeble system has been widely employed in the research of material constitutive model [14,15]. It mainly includes three parts: heating system, mechanical system, and computer control system. The heating system forms a current loop with a loaded metal sample (as a resistor) to heat the metal sample. The heating rate and heating temperature are varied by controlling the current in the sample. Therefore, the Gleeble system is generally unable to measure the high-temperature mechanical properties of non-conductive materials, such as ceramic materials. In order to realize the high-temperature mechanical measurement of non-conductive materials on the Gleeble simulator, we designed and developed an auxiliary thermal device that could expand the measurement material range of the Gleeble simulator [16]. The schematic diagram of the auxiliary thermal system of the Gleeble testing is shown in Figure 2. The measured temperature can reach 1600 °C. The temperature control accuracy is ±4 °C.

## 3. Results and Discussion

### 3.1. High-Temperature Mechanical Properties

**Figure 3:**
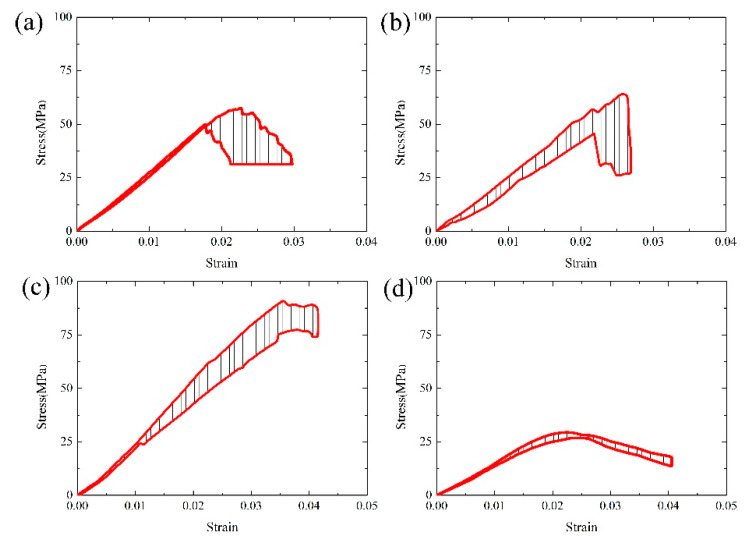
Stress–strain experimental data zone of isothermal uniaxial compression tests: (**a**) ST25, (**b**) ST700, (**c**) ST1100, and (**d**) ST1400.

**Figure 4:**
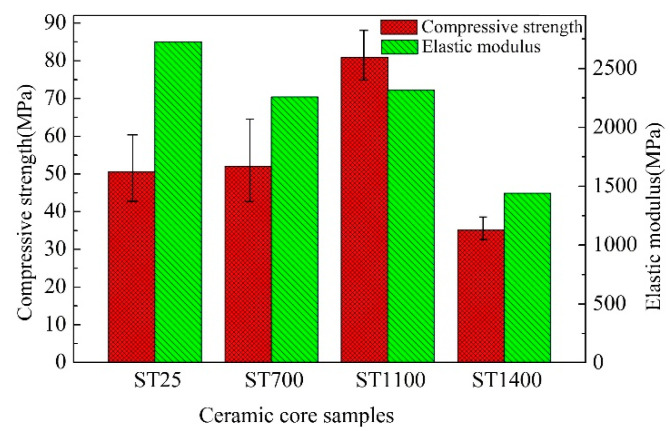
Compressive strength and elastic modulus of samples (ST25, ST700, ST1100, and ST1400).

Figure 3 shows the stress–strain curves variation ranges of the samples ST25, ST700, ST1100, and ST1400, from which can be found ST25, ST700, and ST1100 are all brittle fractures, while ST1400 shows thermo-viscoelastic and viscoplastic property. The average compressive strengths of ST25, ST700, and ST1100 are 51.83, 55.82, and 80.46 MPa, respectively, as shown in Figure 4. It is worth noting that the compressive strength of ST1100 is higher than that of ST25 and ST700. This is mainly due to the conversion of α-cristobalite to β-cristobalite. The densities of α-cristobalite and β-cristobalite are about 2.32 and 2.22 g/cm^3^, respectively, and the expansion of the β-cristobalite volume makes the sample more compact [17]. More α-cristobalite is transformed into β-cristobalite at 1100 °C, and the strength of β-cristobalite is stronger than that of α-cristobalite [18]. At the same time, Zener pinning would be more pronounced at higher temperatures [9].

In the elastic stage, the stress–strain curve of sample ST25 has a good linear-elastic regime. With the increase of temperature, the stress–strain curves of ST700 and ST1100 have a large amplitude and demonstrate a certain degree of dispersion. The elastic moduli of ST25, ST700, ST1100, and ST1400 are 2726, 2259, 2316, and 1442 MPa, respectively. The elastic modulus of the SiO_2_-based ceramic core shows a decreasing trend when the temperature is increased. There is a small change in the range of 25~1100 °C, while the elastic modulus decreases rapidly at the range of 1100~1400 °C. The stress–strain curve of ST1400 at the viscoplastic stage is narrow, indicating that the high-temperature experimental result achieves high repeatability and reproducibility. However, the overall high-temperature mechanical property of the sample ST1400 decreases significantly.

On the other hand, it can be found from Figure 4 that the dispersion properties of compressive strengths vary greatly with the increase of temperature. The samples ST25 and ST700 demonstrate large dispersion, while samples ST1100 and ST1400, especially sample ST1400, show less dispersion. To quantify this result, a universal empirical model called Weibull approach is introduced. The three-parameter Weibull distribution function can be simplified to a two-parameter Weibull distribution function without affecting its accuracy [10,19]:

(1) $$P\left(x\right)=1-\exp\left[-{\left(\sigma /\sigma_{0}\right)}^{m}\right]$$

where P(x) is the failure probability, $\sigma_{0}$ is shape factor, and m is Weibull modulus. Generally speaking, the larger *m* indicates the material is more uniform and less dispersion.

**Figure 5:**
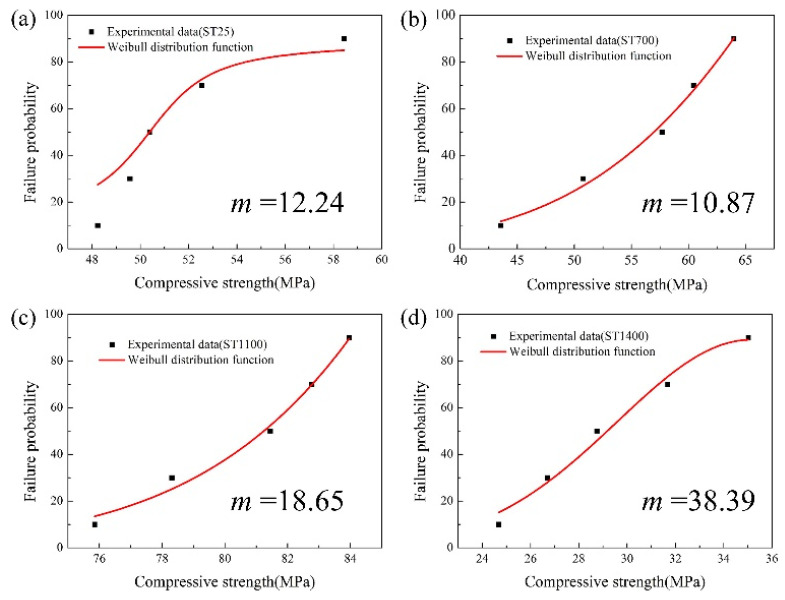
Weibull distribution of high-temperature compressive strengths for SiO_2_ ceramic cores: (**a**) ST25, (**b**) ST700, (**c**) ST1100, and (**d**) ST1400.

From Figure 5, it can be found the Weibull moduli of ST25, ST700, ST1100, and ST1400 are 12.24, 10.87, 18.65, and 38.39, respectively. Obviously, ST1400 demonstrates the largest modulus, indicating that the hot deformation of sample at 1400 °C tends to exhibit certain stable and repeatable characteristic. At the same time, as the moduli of ST700 and ST25 are relatively small, it can be concluded that the deformation at 700 and 25 °C will be quite unstable. In fact, these results furtherly confirm the difference of stress–strain curves in Figure 3 from the angle of data.

### 3.2. Microstructures Evolution

**Figure 6:**
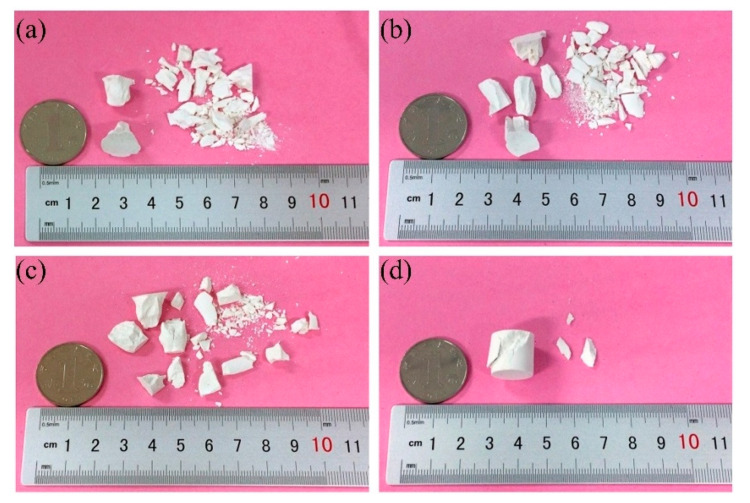
Macrostructural investigation of the sample fracture: (**a**) ST25, (**b**) ST700, (**c**) ST1100, and (**d**) ST1400.

As is well-known, the mechanical properties are always associated with microstructures evolution. Figure 6 exhibits the macrostructural investigations of the samples (ST25, ST700, ST1100, and ST1400) fracture surfaces. The difference in the fracture patterns of ST25, ST700, ST1100, and ST1400 can be clearly distinguished (Figure 6a–d). ST25, ST700, and ST1100 are crushed brittle fractures. As the temperature increases, the average size of residual pieces increases. When the strain of ST1400 reaches 0.04, the sample can maintain a substantially complete shape, and only minor fragments occur on the cylindrical surface. At the same time, a sliding plane of approximately 45° with the bottom surface of the cylindrical sample can be clearly seen. It is this kind of viscous slip that causes the stress plateau of ST1400 after the strain is greater than 0.02 at 1400 °C. The main reason of this sticky slip can be explained in the microstructure morphology.

**Figure 7:**
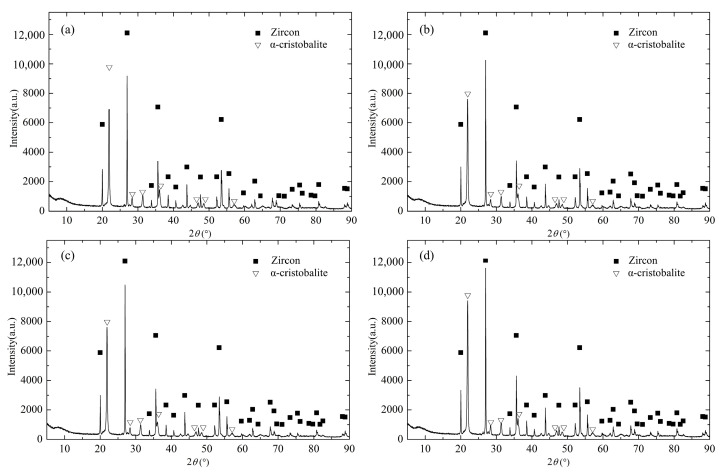
XRD patterns of ceramic samples: (**a**) ST25, (**b**) ST700, (**c**) ST1100, and (**d**) ST1400.

**Figure 8:**
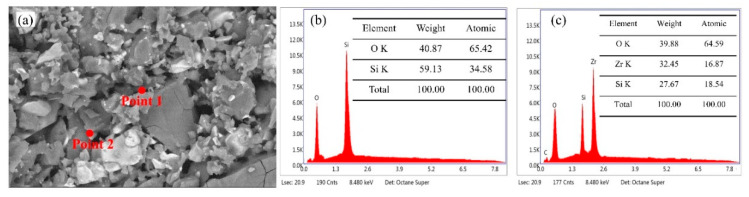
EDS analysis of ST1100: (**a**) the image of BSE, (**b**) EDS result of point 1, and (**c**) EDS result of point 2.

Figure 7 shows the XRD patterns of various samples after hot compression at different temperatures. It can be found that all samples are composed of zircon and α-cristobalite. With the increase of deformation temperature, the peak intensities of two phases have a little change. In order to clarify the phase distribution in the microstructure, EDS point elemental analysis was employed and the results are shown in Figure 8. As shown in Figure 8, the point 1 with gray-black color is SiO_2_ and the point 2 with bright white color is ZrSiO_4_ in the image of backscattered electron (BSE).

**Figure 9:**
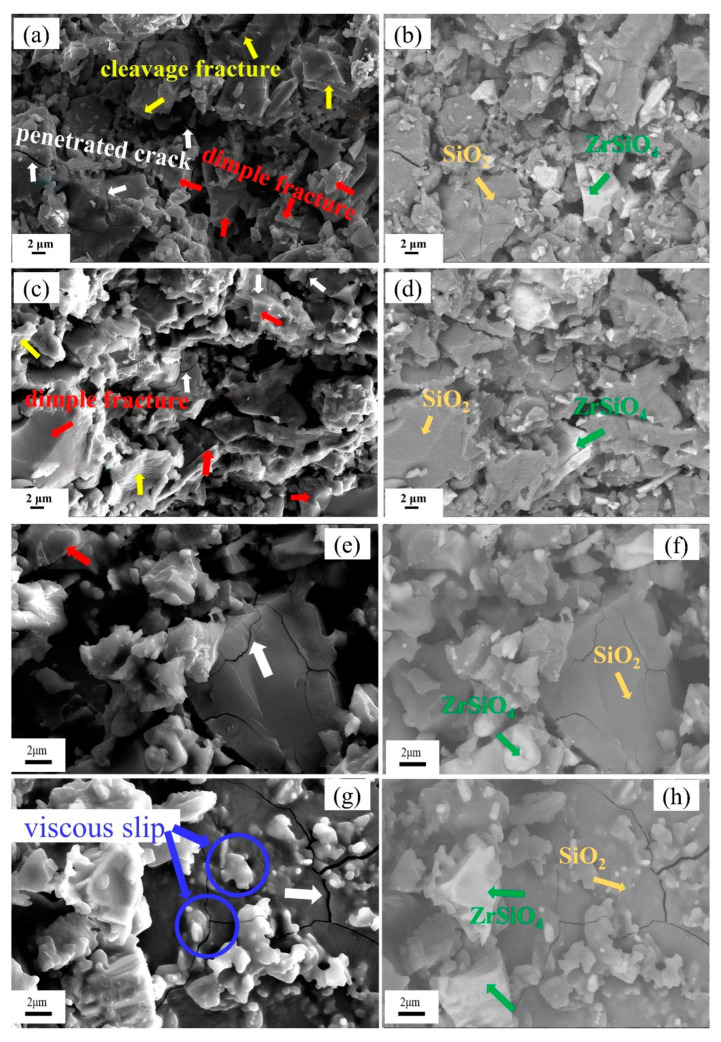
SEM images of the sample fracture surface (red arrows = dimple fracture; yellow arrows = cleavage fracture; blue arrows = high temperature viscous slip; white-gray = ZrSiO_4_, and black-gray = SiO_2_). SE: (**a**) ST25, (**c**) ST700, (**e**) ST1100, and (**g**) ST1400. BSE: (**b**) ST25, (**d**) ST700, (**f**) ST1100, and (**h**) ST1400.

Figure 9a,c,e,g shows the images of secondary electrons (SE), and Figure 9b,d,f,h shows the images of the BSE. In the BSE images, the major component of the white-gray phase is ZrSiO_4_, and the major component of the black-gray phase is SiO_2_. From the micro-topography of ST25, ST700, and ST1100, it can be seen that the SiO_2_ particles mainly undergo cleavage fracture, the ZrSiO_4_ particles mainly undergo dimple fracture. Most penetrated cracks are distributed in larger SiO_2_ particles. However, there are almost no cleavage fractures in SiO_2_ particles of the ST1400, and there are little dimple-like ZrSiO_4_ sections. The surfaces of the small SiO_2_ particles have a smooth curved shape. The occurrence of material fracture usually has great uncertainly, which is also the reason for the divergence of the stress–strain curves. As mentioned before, the Weibull modulus of ST1400 is much larger than that of ST25, ST700, and ST1100, meaning that the deformation of ST1400 is more stable. Therefore, the microstructure observation results of samples above are relatively consistent with the stress–strain curves.

In the cross-section of ST25, ST700, and ST1100, the surfaces of the large SiO_2_ particles are clean, and almost no adhesion of fine SiO_2_ particles is observed. However, in addition to penetrating cracks on the surface of the large SiO_2_ particles in ST1400, a large number of smooth and fine SiO_2_ particles are attached to the surface (Figure 9h). It is generally believed that the melting temperature of β-cristobalite is 1720 ± 10 °C [17,20]. However, some studies have shown that, when the temperature reaches 1400~1450 °C, it will slowly melt on the surface of the SiO_2_, and the presence of other elements or impurities may reduce the β-cristobalite transformation temperature [21,22]. Therefore, in the high-temperature environment of 1400 °C, the main reason for the viscous slip of the SiO_2_-based ceramic core samples is that the surfaces of the fine SiO_2_ particles are initially melted, which plays a role in lubrication between large particles. The SiO_2_, which is initially melted at the temperature of 1400 °C, adheres to the surface of the large SiO_2_ particle. When the temperature drops further to the room temperature, it combines with the large particles, to form a unitary body.

### 3.3. Nonlinear Constitutive Models for High-Temperature Compressive Damage

It can be seen from Figure 4 and Figure 5 that the compressive strength and modulus are basically negatively correlated with temperature. The macro-effect of temperature on the properties of ceramic core includes two aspects. On the one hand, the intermolecular forces decrease with increasing temperature. On the other hand, the change of structure caused by the variation of temperature will greatly affect the properties of the material, such as thermal mismatch. Therefore, the thermal damage *D(T)* is employed to describe the temperature effect on the property [23]:

(2) $$D\left(T\right)=1-E_{T}/E_{0}$$

where $E_{0}$ is elastic modulus at room temperature, and $E_{T}$ is the elastic modulus at *T*. The elastic modulus denoted by thermal damage is expressed as follows:

(3) $$E_{T}=\left[1-D\left(T\right)\right]E_{0}$$

**Figure 10:**
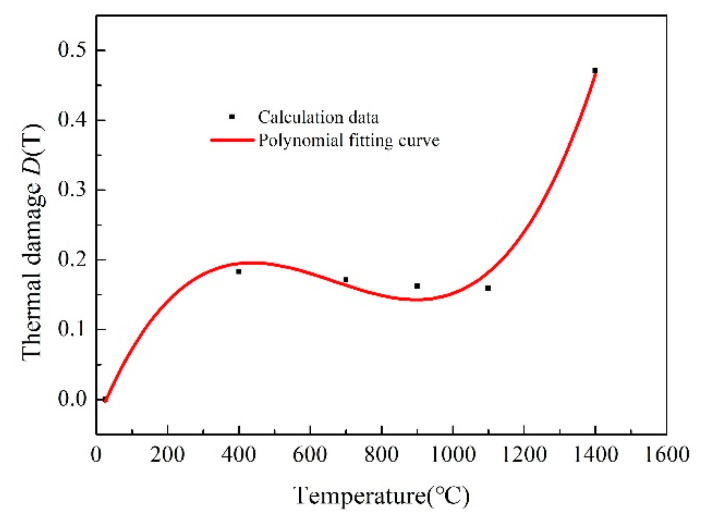
Thermal damage values of ceramic cores under different temperatures.

According the analysis of the experimental results at different temperatures, the thermal damage value, *D(T)*, at each temperature point is calculated, as shown in Figure 10. Through data fitting, the expression of thermal damage with temperature variation can be written as follows:

(4) $$D\left(T\right)=-0.0328+0.00125T-2.136\times 10^{-6}T^{2}+1.068\times 10^{-9}T^{3}$$

According to the author’s previous research [24], the continuous damage constitutive model based on Weibull distribution method, at room temperature, is summarized as follows:

(5) $$\sigma_{1}=E\varepsilon_{1}\exp\left[-{\left(\frac{\varepsilon_{1}}{\varepsilon_{0}}\right)}^{m}\right].$$

where $\varepsilon_{1}$ is the axial strain, and $\varepsilon_{0}$ is a constant. In order to obtain the nonlinear constitutive model for high-temperature compressive damage, the *E* in Equation (5) can be substituted by $E_{T}$, and the formula can be rewritten as follows:

(6) $$\sigma_{1}=\left[1-D\left(T\right)\right]E_{0}\varepsilon_{1}\exp\left[-{\left(\frac{\varepsilon_{1}}{\varepsilon_{0}}\right)}^{m}\right]$$

**Figure 11:**
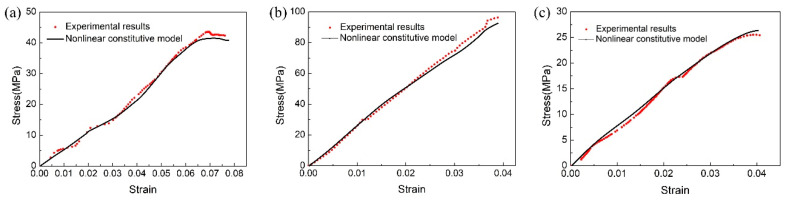
The comparison between the experimental results and nonlinear constitutive model prediction: (**a**) ST700, (**b**) ST1100, and (**c**) ST1400.

The experiment results of typical compression stress–strain of SiO_2_-based ceramic core and the simulation results based on thermo-visco damage model are presented in Figure 11.

From Figure 11, it can be found that the nonlinear constitutive model has a good generalization property. In other words, this model could reflect the uniaxial compression behaviors of ceramic cores deformed at various temperatures.

## 4. Conclusions

(1) In the temperature range from 25 to 1400 °C, the elastic moduli of the SiO_2_-based ceramic cores range from 1442 to 2726 MPa at the elastic stages. The statistical results of Weibull moduli show that the stability of deformation increases with the increase of temperature.
(2) The SiO_2_-based ceramic core samples are all brittle fractures, while, when the temperature exceeds 1400 °C, the mechanical behaviors of the samples are characterized by thermo-viscoelastic and viscoplastic properties, which mainly can be ascribed to the initial surface melting of SiO_2_ fine particles.
(3) Nonlinear constitutive model for high-temperature compressive damage is established to predict the hot deformation of ceramic core. The comparison results between the nonlinear model predictions and experimental values indicate that the model is applicable.

## Figures and Tables

**Table 1:** Characteristics of the used fused silica and zircon as raw materials.

Powder (%)	SiO_2_	ZrO_2_	Al_2_O_3_	K_2_O	CaO	TiO_2_	Powder Density (g/cm^3^)	Open Porosity (%)	Source
Fused Silica	99.99	-	0.002	-	0.004	0.001	1.99	0.8	HongDa
Zircon	33.21	62.5	0.82	0.90	0.55	1.94	4.54	1.1	XinTai

**Table 2:** Heat treatments of ceramic cores to simulate the directional solidification process and test conditions.

Ceramic Core Samples	Sintering	Heat Treatment	Test Temperature
ST25	1000 °C @ 60 min	-	25 °C
ST700	1000 °C @ 60 min	1500 °C @ 30 min	700 °C
ST1100	1000 °C @ 60 min	1500 °C @ 30 min	1100 °C
ST1400	1000 °C @ 60 min	1500 °C @ 30 min	1400 °C
